# Associations between Urinary, Dietary, and Water Fluoride Concentrations among Children in Mexico and Canada

**DOI:** 10.3390/toxics8040110

**Published:** 2020-11-20

**Authors:** Rivka Green, Christine Till, Alejandra Cantoral, Bruce Lanphear, E. Angeles Martinez-Mier, Pierre Ayotte, Robert O. Wright, Martha M. Tellez-Rojo, Ashley J. Malin

**Affiliations:** 1 Department of Psychology, York University, Toronto, ON M3J 1P3, Canada; rrgreen@yorku.ca (R.G.); ctill@yorku.ca (C.T.); 2 Departamento de Salud, Universidad Iberoamericana, Ciudad de México 01219, Mexico; 3 Faculty of Health Sciences, Simon Fraser University, Vancouver, BC V5A 1S6, Canada; bpl3@sfu.ca; 4 School of Dentistry, Indiana University, Indianapolis, IN 46202, USA; esmartin@iu.edu; 5 Department of Social and Preventive Medicine, Laval University, Quebec City, QC G1V 0A6, Canada; pierre.ayotte@inspq.qc.ca; 6 Environmental Medicine and Public Health, Icahn School of Medicine at Mount Sinai, New York, NY 10029, USA; robert.wright@mssm.edu (R.O.W.); ashley.malin@mssm.edu (A.J.M.); 7 School of Public Health, Instituto Nacional de Salud Pública, Cuernavaca 62100, Mexico; mmtellez@insp.mx

**Keywords:** urinary fluoride, childhood, Mexico, Canada

## Abstract

Fluoride, which may be toxic to the developing brain, is added to salt in Mexico and drinking water in Canada to prevent dental caries. We compared childhood urinary fluoride (CUF) concentrations in Mexico City and Canada to characterize patterns of fluoride exposure in these two populations. We also examined associations of CUF with dietary and water fluoride levels in Mexico City and Canada respectively. We included 561 children (ages 4–6; mean age 4.8 years) from the Programming Research in Obesity, Growth, Environment, and Social Stress (PROGRESS) cohort in Mexico City, and 645 children (ages 2–6; mean age 3.7 years) from the Maternal–Infant Research on Environmental Chemicals (MIREC) cohort in Canada. We applied Spearman correlations, T-tests, ANOVA or covariate-adjusted linear regression to examine associations of CUF (mg/L; adjusted for specific gravity) with demographics and dietary or water fluoride concentrations. We used Welch equivalence testing to compare means across cohorts. Mean (SD) CUF was equivalent (*t* = 4.26, *p* < 0.001) in PROGRESS: 0.74 (0.42) and fluoridated Canadian communities: 0.66 (0.47), but lower in non-fluoridated Canadian communities: 0.42 (0.31) (*t* = −6.37, *p* < 0.001). Water fluoride concentrations were significantly associated with CUF after covariate adjustment for age and sex in MIREC (B = 0.44, 95% CI: 0.30, 0.59, *p* < 0.001). In contrast, daily food and beverage fluoride intake was not associated with CUF in PROGRESS (*p* = 0.82). We found that CUF levels are comparable among children in Mexico City and fluoridated Canadian communities, despite distinct sources of exposure. Community water fluoridation is a major source of fluoride exposure for Canadian children.

## 1. Introduction

Fluoride is often added to water, salt, or milk to prevent dental cavities [1]. Approximately 39% of Canadians and 73% of Americans on public water distribution systems have fluoride added to drinking water to achieve the optimal concentration of 0.7 mg/L for dental carries prevention [2,3]. In the United States (US), fluoridated water is the main source of fluoride exposure, accounting for approximately 60% of total daily fluoride intake among adults, and 42% of total daily fluoride intake among children under the age of 12 [4]. In Mexico, sodium fluoride or potassium fluoride are added to salt at 250 ± 50 mg/L in regions where naturally occurring fluoride in water is less than 0.7 mg/L, such as Mexico City [5]. Other sources of fluoride can include fluoridated dental products and supplements, certain dietary products that contain naturally occurring fluoride, such as green and black tea, and foods that are sprayed with fluoride-containing pesticides (i.e., grapes) [6,7].

Water fluoride concentrations are moderately to strongly correlated with urinary fluoride levels [8,9,10] and blood plasma levels [11,12]. Indeed, urinary fluoride levels typically approximate the concentration of fluoride found in tap water in adults [9,13,14] and children [15,16]. In Mexico, salt fluoride intake patterns or contributions of salt fluoride to urinary fluoride levels are not well-established [17].

Fluoride may be a developmental neurotoxicant, even in populations exposed to “optimal” levels, especially for exposure in the prenatal period [18,19,20,21,22,23]. For example, cross-sectional studies have reported associations between higher water fluoride levels in Canada and an increased risk of ADHD symptoms and/or diagnosis in children and adolescents, but they did not find the same association with child urinary fluoride [18]. Similarly, most recent studies of cohorts exposed to optimally fluoridated levels have not found an association between fluoride exposure in childhood and adverse neurodevelopmental outcomes, such as diminished IQ scores and diagnosis of a learning disability or ADHD [15,22,24,25].

There are developmental factors that can influence urinary fluoride concentration in children. For example, fluoride excretion is lower in children relative to adults due to differences in absorption [12,26]; children younger than seven years retain approximately 55–61% of daily ingested fluoride compared with 36% in healthy adults [7,27]. Further, children younger than four years of age are more likely to swallow toothpaste due to an immature swallowing reflex, which contributes to increases in blood fluoride concentrations [28] and higher total daily fluoride intake [7]. Given widespread exposure to fluoride and the fine balance between optimal levels for oral health and excessive levels that may contribute to neurotoxicity, it is prudent to monitor fluoride intake and exposure levels among young children in North America.

Prior reports and Canadian studies that have characterized childhood urinary fluoride (CUF) have done so irrespective of fluoridation status [13,29]. For example, the Canadian Health Measures Survey (CHMS) reports a median creatinine-adjusted CUF level of 0.79 mg/g (unadjusted median CUF of 0.51 mg/L) among children ages 3–5 years across Canada [29]. However, it is important to consider fluoridation status because urinary fluoride levels are approximately 1.5–2 times higher in fluoridated regions than non-fluoridated regions [9,30,31]. The purpose of this study was to compare child urinary fluoride (CUF) concentrations with dietary and water fluoride levels among children in Mexico City and Canada respectively. We also compared CUF levels by age, sex, and other demographic variables, within these two populations.

## 2. Materials and Methods

### 2.1. Study Sample

MIREC: The Maternal–Infant Research on Environmental Chemicals (MIREC) study is a large sample of pregnant women and children that were recruited from ten cities across Canada from 2008–2011 [32]. A subset of 2–6-year-old children from MIREC (*N* = 654) were recruited for the biomarker visit (MIREC-CD Plus) in childhood; of these, 645 (98.6%) had CUF samples and 516 (78.9%) had water fluoride measurements. A total of 450 (68.8%) children had both a CUF sample and a water fluoride value, as well as complete covariate data. Ethics approval for MIREC was received from Health Canada’s Research Ethics Board (Project #: 2016-0015; approval date: 25 July 2016), York University Research Ethics Board, and Indiana University’s Ethics Board. Informed consent was received from all participants.

PROGRESS: The Programming Research in Obesity, Growth, Environment, and Social Stress (PROGRESS) study is an ongoing NIH-funded, prospective birth cohort in Mexico City that involves 948 mother–offspring pairs who are followed from the 2nd trimester of pregnancy to the child’s 10th birthday. Children are followed every six months following birth and every two years after 24 months of age. Multiple biological matrices (blood, plasma, hair, nails, urine, saliva) were collected longitudinally from mothers and their children. A more detailed description of the PROGRESS participant recruitment process is described elsewhere [33,34]. CUF and food/beverage fluoride values were analyzed among 551 and 561 participants, respectively. Participants in PROGRESS were eligible for inclusion in regression analyses if they had CUF measurements, complete covariate data, and food/beverage fluoride intake estimates (*n* = 539). Institutional Review Board approval for the PROGRESS study was granted by The Icahn School of Medicine at Mount Sinai (Project #12-00751A; approved for continuation: 7 July 2020), Harvard School of Public Health, The National Institute of Public Health in Mexico (Project #: CI-560-13072012; approval date: 13 July 2012), and the Brigham and Women’s Hospital.

### 2.2. Childhood Urinary Fluoride (CUF)

We collected single urine samples under non-fasting conditions. For both the PROGRESS and MIREC cohorts, CUF concentrations were analyzed at the Oral Health Research Institute at Indiana University using a modification of the hexamethyldisoloxane (HMDS; Sigma Chemical Co.) microdiffusion procedure as modified by Martinez-Mier et al. (2011) [35,36]. A measured and recorded volume of each sample (0.850 mL) was dispensed into 15-cm plastic Petri dishes (Falcon Fisher Scientific Co.); a sodium hydroxide (NaOH, anhydrous; Sigma Chemical Co.) trap solution was loaded onto the Petri dish lid and after adding sulfuric acid (H_2_SO_4_; Sigma Chemical Co.) saturated with HMDS, each dish was tightly sealed. Fluoride was released by acid hydrolysis and trapped in the NaOH trap. The fluoride-containing trap was then removed and buffered to pH 5.2 with perchloric acid (HClO_4_; Sigma Chemical Co.). The resulting solution was adjusted to a final volume of 100 µL with total ionic strength buffer (TISABII; Fisher Scientific Co.). Fluoride levels were determined by comparing the millivolt reading of each sample to standard curves, covering the range of the samples’ values. Reference standard solutions were monitored daily by a quality assurance (QA) officer for stability.

To account for variations in urine dilution, CUF concentrations were standardized for specific gravity (SG) using the following equation [37]: CUF_SG_ (mg/L) = CUF*i* * (SG*m* − 1)/(SG*i* − 1) where CUF_SG_ (mg/L) is the SG standardized fluoride concentration, CUF*i* is the observed fluoride concentration, SG*i* is the SG of the individual urine sample and SG*m* is the median SG for the sample. Detailed methodology for urinary fluoride analysis and SG standardization are reported elsewhere [9].

### 2.3. Dietary Fluoride Concentration (for the PROGRESS Cohort Only)

Validated food frequency questionnaires (FFQ) were administered to mothers in PROGRESS to assess dietary patterns of their children over the previous week. FFQs included 101 foods/beverages grouped into 14 categories [38]. Total food and beverage fluoride intake (mcg/d) was calculated for 561 children using the estimates previously reported [17]. These estimates are based on analyses of fluoride content in 182 foods or beverages from Mexico City Markets and Supermarkets [17]. Foods were homogenized, and then frozen aliquots were shipped to the Indiana University School of Dentistry Oral Health Research Institute for fluoride analysis. Analysis of food and beverages was conducted using a modification of the hexamethyldisiloxane (HMDS: Sigma-Aldrich, Indianapolis, IN, USA) microdiffusion method as modified by Martinez-Mier et al. as described elsewhere [35].

### 2.4. Measurement of Municipal Drinking Water Fluoride Levels (for the MIREC Cohort Only)

We linked municipal public water reports with the first three digits of participants’ postal codes, and water boundary regions were predetermined for each city. Water fluoride levels were only derived for those participants whose mothers reported drinking tap water as the primary drinking water source as a proxy for the type of water that children drink. For each participant, water fluoride values represented the average fluoride concentration measured by the water treatment plants over the six months prior to the urine sample.

Although naturally occurring water fluoride may exist in some of the non-fluoridated areas included in the study, the mean concentrations of fluoride in these regions is approximately 0.1 mg/L. The present study defined a non-fluoridated site as having water fluoride levels (both adjusted and natural fluoride levels) as <0.3 mg/L. All fluoridated sites had corresponding water treatment plants that reported adding fluoride to the water supplies according to national drinking water guidelines [39], which includes a recommended range of 0.6 mg/L to 0.8 mg/L fluoride in the water.

### 2.5. Covariates and Modifiers of Exposure Levels

We selected variables that may modify fluoride metabolism and excretion based on literature review [26,40]. We examined the following covariates: age, sex, maternal education, body mass index (BMI; PROGRESS cohort), and child weight (MIREC cohort). Maternal education was defined as obtaining less than a secondary school education, a secondary school education, or greater than a secondary school education in the PROGRESS cohort, whereas in the MIREC cohort, given that the majority of the women were university educated, it was defined as university degree or higher versus college degree or lower. We also assessed race (white vs. non-white) in the MIREC cohort, but not for the PROGRESS cohort because of the limited heterogeneity in race and ethnicity.

### 2.6. Statistical Analyses

We used Spearman correlations to assess associations of CUF_SG_ concentrations with age and BMI (in PROGRESS) or weight (in MIREC). We used t-tests or ANOVA to test mean differences in CUF_SG_, food/beverage fluoride (for PROGRESS) or water fluoride (for MIREC), according to sex or maternal education, as well as race in MIREC. We used partial correlations to assess associations of food/beverage fluoride with age in PROGRESS, controlling for weight or total daily caloric intake. We applied two one-sided tests (TOST) Welch equivalence testing [41] to compare CUF_SG_ means between PROGRESS and MIREC, which allows for skewed outcome data and does not assume equal variances between groups [42]. We conducted multiple linear regression to examine associations of CUF_SG_ with food/beverage fluoride concentrations in PROGRESS and water fluoride concentrations in MIREC adjusted for covariates. We considered potential covariates a priori including age, sex, BMI/weight, race (MIREC only), and maternal education. However, we only retained covariates in the model that changed the B coefficient by 10% and/or had a *p* < 0.20 when included in the model. The covariates that met criteria for inclusion in the regression models were age, sex and maternal education for PROGRESS, and age and sex for MIREC. In PROGRESS, we applied a log_10_-transformation of CUF_SG_ and food/beverage fluoride intake prior to regression analysis to correct for skewness in CUF_SG_ and food/beverage fluoride variables as well as to help to satisfy linear regression assumptions. In both cohorts, assumptions of linear regression were satisfied; no issues with multicollinearity were identified according to variable inflation factors, and no outliers were identified according to Cook’s distance estimates. We also tested interactions of CUF_SG_*sex and CUF_SG_*age in both cohorts to assess whether the exposure-response profiles differed by sex or age. In MIREC, we tested the binary interaction of CUF_SG_*age (i.e., 2–3-year olds versus 4–6-year olds) to allow for more direct comparisons with PROGRESS, while in PROGRESS we included age as a continuous variable. These interaction terms were only retained in models for which they were significant. Two-tailed alphas of 0.05 for main effects and two-tailed alphas of 0.1 for interactions were the criteria for statistical significance in regression analyses.

## 3. Results

Demographic characteristics are presented in Table 1. The male to female ratio was balanced in both cohorts (i.e., approximately 50:50). The average age of children in the PROGRESS and MIREC cohorts was 4.80 and 3.66 years, respectively. The majority of mothers in MIREC (70.9%) had an undergraduate degree or higher, while most mothers in PROGRESS (76.1%) obtained a secondary school education or less. In MIREC, 87.4% of children were described as white by their mothers.

Descriptive statistics for CUF_SG_ as well as dietary fluoride in PROGRESS and water fluoride in MIREC are presented in Table 2. CUF_SG_ was statistically equivalent (*t* = 4.26, *p* < 0.001) in PROGRESS (M = 0.74, SD = 0.42) and fluoridated Canadian communities (M = 0.66, SD = 0.47), but lower in non-fluoridated Canadian communities (*M* = 0.42, *SD* = 0.31), as compared to fluoridated Canadian communities (*t* = −6.37, *p* < 0.001). The equivalence test comparing CUF_SG_ in PROGRESS to the 4–6-year olds specifically from fluoridated communities in MIREC was also significant (*t* = −3.47, *p* < 0.001).

### 3.1. Association between CUF_SG_ and Covariates

CUF_SG_ was weakly and positively correlated with age in PROGRESS (ρ = 0.10, *p* = 0.02) and MIREC (ρ = 0.23, *p* < 0.001). In MIREC, CUF_SG_ was similar in males (M = 0.57, SD = 0.43) and females (M = 0.53, SD = 0.42; *t* = 1.14, *p* = 0.25); no sex differences were observed in PROGRESS either (*t* = 0.04, *p* = 0.97). We did not observe significant differences in CUF_SG_ according to race in MIREC (*t* = −1.42, *p* = 0.16). CUF_SG_ was not associated with maternal education in PROGRESS (*F* = 0.75, *p* = 0.47) or MIREC (*t* = −1.45, *p* = 0.15). CUF_SG_ was not correlated with BMI in PROGRESS (ρ = −0.02, *p* = 0.73) or child weight in MIREC (ρ = 0.10, *p* = 0.20).

### 3.2. Association between Fluoride Sources and Covariates

In PROGRESS, dietary fluoride intake was weakly and positively associated with age (*r* = 0.17, *p* < 0.001) after adjusting for weight, such that older children consumed more fluoride from their daily diet than younger children. However, the association between dietary fluoride and age was attenuated after controlling for total daily calorie intake (*r* = 0.08, *p* = 0.07). There were no significant differences in food/beverage fluoride intake according to maternal education (*F* = 1.62, *p* = 0.20) or sex (*t* = 1.1, *p* = 0.27) and food/beverage fluoride intake was not associated with BMI (ρ = −0.02, *p* = 0.59).

In MIREC, as an artifact of the study design where sites began recruitment at different times, we observed that older children lived in areas with higher water fluoride concentrations; the correlation between water fluoride concentration and age was (ρ = 0.18, *p* < 0.001). There were also significant differences in water fluoride concentration according to maternal education and race. Mothers who were less educated (i.e., college degree or lower) were more likely to live in cities with lower water fluoride concentrations (M = 0.30 mg/L, SD = 0.24) than mothers with a higher education level (i.e., bachelor’s degree or higher, M = 0.36 mg/L, SD = 0.26, *t* = 2.43, *p* = 0.02). Additionally, non-white mothers were somewhat more likely to live in cities with lower water fluoride levels (M = 0.30 mg/L, SD = 0.25) than white mothers (M = 0.35 mg/L, SD = 0.25), but this difference did not reach significance (*t* = −1.55, *p* = 0.12). There were no significant differences in water fluoride concentrations by child sex (*t* = 0.15, *p* = 0.88) and water fluoride concentration was not associated with child weight (ρ = 0.04, *p* = 0.59).

### 3.3. CUF in Relation to Fluoride Sources

**Figure 1:**
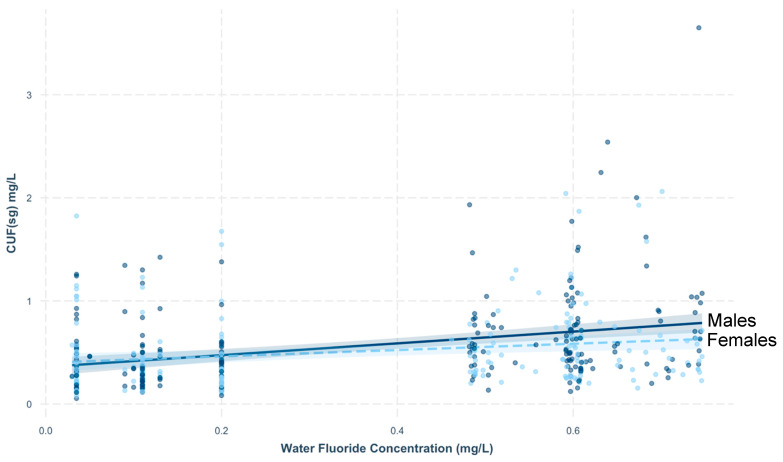
Associations of water fluoride concentration with CUF_SG_ concentration in the MIREC cohort according to sex.

Daily dietary fluoride intake was not associated with CUF_SG_ in PROGRESS (B = −0.01, 95%, CI: −0.06, 0.05, *p* = 0.82). In MIREC, water fluoride concentration was significantly associated with CUF_SG_, after adjustment for age and sex (B = 0.44, 95%, CI: 0.30, 0.59, *p* < 0.001); this association significantly interacted with sex (*p* = 0.07), but not age (*p* = 0.20). Specifically, water fluoride levels tended to be more strongly associated with CUF_SG_ levels for males (B = 0.57, 95% CI: 0.37, 0.77), as compared to females (B = 0.31, 95% CI: 0.10, 0.51; Figure 1).

## 4. Discussion

We characterized child urinary fluoride (CUF_SG_) levels in two large prospective pregnancy and birth cohorts from Mexico (PROGRESS) and Canada (MIREC) to compare CUF_SG_ levels as a function of fluoride sources and sociodemographic factors. Even though fluoride is added to salt in Mexico and water in some cities in Canada, we observed equivalent mean CUF_SG_ concentrations among children in both cohorts. As expected, urinary fluoride levels were lower among children who lived in non-fluoridated (*M* = 0.42 mg/L, *SD* = 0.31) than in fluoridated communities (*M* = 0.66 mg/L, *SD* = 0.47, Table 2).

Our findings are consistent with past biomonitoring studies conducted in children showing that levels of urinary fluoride are approximately 1.5 to 2 times higher in fluoridated regions than in non-fluoridated regions [9,31]. However, another study in Brazil [43] did not find meaningful differences in CUF_SG_ patterns in relation to water fluoride levels among children ages 2–7-year olds. Still, all of the children in that study were exposed to water fluoride levels higher than 0.5 mg/L and fluoride retention has been shown to plateau at higher total daily fluoride intake levels [7].

We observed similar patterns of fluoride exposure by demographic factors in the two cohorts. In both PROGRESS and MIREC, CUF levels tended to increase with age. Interestingly, in PROGRESS we found that older children tended to consume more fluoride from their daily diet than younger children, even after controlling for weight; although, this association was partially due to higher caloric intake among older children. In MIREC, due to an artifact of study design, water fluoride was slightly correlated with age such that older children tended to live in regions with higher water fluoride concentrations. Thus, fluoride exposure or intake from community fluoridation (i.e., salt in Mexico and water in Canada) may partially account for differences in biomarker fluoride levels among children according to age. Older children are also more likely to use fluoridated toothpaste than 2–3-year-old children [28].

In MIREC, CUF and water fluoride concentrations did not differ according to sex. Consistently, in PROGRESS we did not observe differences in CUF or dietary fluoride intake by child’s sex. We also did not find meaningful associations between CUF or dietary/water fluoride with BMI (in PROGRESS), weight (in MIREC), maternal education or race (in MIREC), although most (87%) of the MIREC sample was white.

Dietary fluoride intake was not associated with CUF in the PROGRESS cohort. The FFQ utilized to estimate dietary fluoride intake did not, however, directly measure salt added to food after preparation. Given that salt fluoridation is widespread in Mexico [44], it is possible that our measure of salt intake was imprecise. Future studies employing more objective measures of dietary and salt fluoride intake should be conducted to more rigorously characterize contributions of dietary fluoride intake to CUF in Mexico.

In the MIREC cohort, we found that children exposed to higher water fluoride concentrations had significantly higher CUF concentrations, especially boys. Specifically, for each 1 mg/L increase in water fluoride concentration in Canada, CUF increased by 0.44 mg/L among all children; CUF increased by 0.57 mg/L among boys. This finding suggests that there may be metabolic differences in fluoride exposure between boys and girls that warrant further exploration. It is also consistent with our prior study which showed that each 1 mg/L increase in water fluoride concentration was associated with a 0.57 mg/L increase in urinary fluoride among pregnant women in Canada [9]. Water fluoride thus appears to be a major source of fluoride exposure for both pregnant Canadian women and their children. Interestingly, mean urinary fluoride levels among children were very similar to those of their mothers (averaged across pregnancy) in both fluoridated (child: 0.66 mg/L; maternal: 0.71 mg/L) and non-fluoridated (child: 0.42 mg/L; maternal: 0.41 mg/L) areas.

Understanding the magnitude, patterns and sources of fluoride exposure among children is important for understanding the potential risk for adverse effects, as well as identifying vulnerable populations.

Strengths of our study include assessment of various sources of fluoride and multiple covariates, large sample sizes, and adjusting for specific gravity to control for varying urinary dilution across both cohorts. This study also has some limitations. First, urinary fluoride concentrations were measured in spot samples and there can be intra-individual variability in urinary fluoride concentrations from daily behaviors that were not controlled in this study (e.g., use of fluoridated toothpaste and consumption of bottled water). Additionally, because fluoride has a relatively short half-life (approximately six hours) [40], urinary fluoride measurements reflect recent exposures. Second, we relied on the mothers’ self-reported use of tap water to estimate child tap water exposure, and we excluded those that did not report drinking tap water as we did not have information on bottled water use. For measurements of water fluoride, water fluoride concentrations were measured at the water treatment plant rather than the tap. However, this is likely to be non-differential misclassification of exposure since we would not expect differences in drinking water reporting based on fluoridation status. Third, the FFQ utilized in this study for PROGRESS may not have adequately captured true dietary fluoride intake because FFQs can be subject to recall biases/forgetting and the FFQs used in this study did not measure added salt.

## 5. Conclusions

We found that urinary fluoride levels are comparable among children in Mexico City and fluoridated Canadian communities, even though their sources of fluoride exposure differ. Other factors, including age and sex may also influence exposure patterns. Community water fluoridation in Canada appears to be a major source of fluoride exposure among children. Further studies are needed to clarify whether higher CUF among older children reflects different sources of exposure and/or differences in toxicokinetics, as well as investigate potential health effects of early-life fluoride exposure.

## Figures and Tables

**Table 1:** Demographic characteristics of children with child urinary fluoride data.

Demographic Variables	MIREC (*n* = 645)	PROGRESS (*n* = 551)
**Child Variables**		
Sex; N (%) male Age (y); M (SD)	326 (50.5)	282 (51.2)
	3.66 (0.87)	4.80 (0.56)
Weight (kg) or BMI (kg/m^2^)	15.82 (1.88) kg	15.71 (1.71) kg/m^2^
**Maternal Variables**		
Education; N (%)		
	≥University Degree; 455 (70.9)	<secondary school; 217 (39.4)
	≤College Degree; 187 (29.1)	secondary school; 202 (36.7)
		>secondary school; 132 (24)
Race (n, % white) ^a^	564, 87.9%	N/A

^a^ data collected for Maternal–Infant Research on Environmental Chemicals (MIREC) study only. BMI = Body Mass Index.

**Table 2:** Descriptive statistics for childhood urinary fluoride (CUF), water fluoride, and dietary fluoride levels in the MIREC and PROGRESS cohorts.

Fluoride Matrix	N	Arithmetic Mean (SD)	Median (IQR)	5th Percentile; 95th Percentile
**CUF_SG_ (mg/L)**				
MIREC: Overall	645 ^a^	0.55 (0.43)	0.43 (0.39)	0.15; 1.35
Fluoridated	219	0.66 (0.47) *	0.53 (0.43)	0.22; 1.58
Non-fluoridated	231	0.42 (0.31) *	0.33 (0.27)	0.12; 1.14
MIREC (2–3-years)				
Fluoridated	140	0.50 (0.39) *	0.50 (0.39)	0.22; 1.47
Non-fluoridated	170	0.41 (0.32) *	0.32 (0.28)	0.12; 1.13
MIREC (4–6 years)				
Fluoridated	79	0.79 (0.56) *	0.64 (0.59)	0.25; 1.63
Non-fluoridated	61	0.46 (0.29) *	0.37 (0.29)	0.17; 1.15
PROGRESS (4–6 years)	551	0.74 (0.42)	0.67 (0.36)	0.30; 1.32
**Water Fluoride** (mg/L)				
MIREC: Overall	516 ^a^	0.35 (0.25)	0.20 (0.49)	0.035; 0.73
Fluoridated	244	0.60 (0.08)	0.60 (0.09)	0.49; 0.74
Non-fluoridated	272	0.12 (0.06)	0.11 (0.17)	0.04; 0.2
**Dietary Fluoride** (mcg/d)				
PROGRESS	561	693.89 (311.67)	630.64 (372.73)	321.27; 1261.15

Note. * *p* < 0.01 for *t*-tests; ^a^ sample sizes do not equal full sample size as not every child with a CUF measurement had a corresponding water fluoride measurement and vice versa.

## Abbreviations

MIREC = Maternal–Infant Research on Environmental Chemicals; CUF = child urinary fluoride; FFQ = Food Frequency Questionnaire; PROGRESS = Programming Research in Obesity, Growth, Environment and Social Stress.
